# Cyclin-Dependent Kinase-9 and Oxidative Phosphorylation Inhibition Overcomes Ibrutinib Resistance in Mantle Cell Lymphoma

**DOI:** 10.1158/2767-9764.CRC-25-0818

**Published:** 2026-05-22

**Authors:** Carly Roleder, Xiaofan Zhao, Vi Lam, Haifeng Shen, Edward C. Dominguez, Canping Chen, Sonia Rodriguez-Rodriguez, Lili Wang, Tycel Phillips, Zheng Xia, Alexey V. Danilov

**Affiliations:** 1 City of Hope National Medical Center, Duarte, California.; 2 Department of Medicine, Columbia University, New York, New York.; 3 Department of Biomedical Engineering, Oregon Health & Science University, Portland, Oregon.; 4 Knight Cancer Institute, Oregon Health & Science University, Portland, Oregon.

## Abstract

Resistance to Bruton tyrosine kinase inhibitors is inevitable in mantle cell lymphoma (MCL). Cyclin-dependent kinase-9 (CDK9), a key regulator of oncogenic transcription, is a promising therapeutic target. In this study, we studied a selective CDK9 inhibitor, AZD4573, in MCL. Treatment with AZD4573 thwarted growth of both parental and ibrutinib-resistant MCL cell lines and primary MCL cells and downregulated expression of MYC and MCL1. However, CDK9 inhibition enhanced basal and maximal oxygen consumption rate, as well as increased production of ATP and reactive oxygen species in ibrutinib-resistant cell lines and primary MCL cells. Whereas treatment with AZD4573 led to modest prolongation of survival in an ibrutinib-resistant MCL patient-derived xenograft mouse model, accompanied by downregulation of TNFα/NF-κB and mTORC1 signaling pathways in murine splenocytes, oxidative phosphorylation (OxPhos) was upregulated, suggesting tumor metabolic reprogramming. Single-cell RNA sequencing analysis of peripheral blood mononuclear cells (PBMC) from patients treated with AZD4573 on a clinical trial demonstrated sustained downregulation of MYC targets and OxPhos in malignant B cells from a responding patient with MCL. Conversely, two refractory patients exhibited upregulation of MYC targets and OxPhos in PBMCs. OxPhos inhibitor IACS-010759 demonstrated synergy with AZD4573 *in vitro*. Thus, CDK9 inhibition exhibits activity in ibrutinib-resistant MCL and can be further enhanced by cotargeting of OxPhos.

**Significance:**

Ibrutinib resistance is a challenge in MCL. Targeting CDK9 partially overcomes this resistance but results in upregulation of cell metabolism contributing to survival. Dual targeting of CDK9 and OxPhos cooperates to suppress tumor growth, providing a rationale for future exploration of combination therapies in MCL.

## Introduction

Mantle cell lymphoma (MCL) is characterized by complex genomic heterogeneity and variable clinical course, but all patients ultimately develop resistance to available therapies (1). Chronic B-cell receptor (BCR) signaling contributes to lymphomagenesis in B-cell non–Hodgkin lymphomas (NHL; ref. 2). Inhibitors of Bruton tyrosine kinase (BTK), a BCR-associated kinase, have become standard in MCL treatment in both relapsed/refractory and first-line settings (3). Several BTK inhibitor (BTKi) regimens are being actively investigated in therapy of *de novo* MCL (4, 5). However, resistance to BTKis is inevitable and is associated with refractoriness to subsequent therapies and poor overall survival (6–8). In particular, presence of *TP53* aberrations predisposes to inferior responses to BTKi therapy (9).

Cyclin-dependent kinases (CDK) are Ser/Thr kinases that associate with specific cyclins during the cell cycle to regulate its progression. Furthermore, a subset of CDKs (termed transcriptional CDKs, e.g., CDK7 and CDK9) regulate mRNA synthesis. CDK9, a component of the positive transcription elongation factor b, facilitates RNA transcription by phosphorylating RNA polymerase II (RNAPII) at the Ser2 site (10). We previously showed that pharmacologic targeting of CDK9 using AZD4573, a selective inhibitor, downmodulates multiple oncogenic proteins, including MYC and MCL1, in diffuse large B-cell lymphoma (DLBCL) cells *in vitro* and *in vivo* (11, 12). However, CDK9 inhibition (CDK9i) also induced reprogramming of the epigenetic landscape and elicited superenhancer-driven recovery of select oncogenes (e.g., *MYC* and *PIM3*), which could ultimately contribute to therapeutic resistance. Whereas CDK9 inhibitors have been investigated in clinical trials in lymphoid malignancies, CDK9i in a setting of BTKi resistance has not been well studied. In this study, we investigated the effects of AZD4573 in ibrutinib-naïve and ibrutinib-resistant MCL models *in vitro* and *in vivo* and in primary cells from patients treated with AZD4573 on a clinical trial.

## Materials and Methods

### Cell culture

MCL cell lines Mino (RRID: CVCL_1872) and JeKo-1 (RRID: CVCL_1865; both harboring *TP53* aberrations) were purchased from the American Type Culture Collection (ATCC). Ibrutinib resistance was induced in Mino and JeKo-1 cell lines by stepwise exposure to increasing concentrations of ibrutinib (denoted as Mino-IR and JeKo-IR). Cells were cultured in RPMI-1640 medium supplemented with 15% FBS, 1% L-Glutamine, 1% nonessential amino acids, and 1.6% HEPES. Stromal cell lines L (ATCC) and L4.5 (DSMZ Braunschweig) were maintained in DMEM medium with 10% FBS. B-cell activating factor (BAFF)-expressing Chinese hamster ovary cells described previously were cultured in MEM-α supplemented with 10% FBS (13). *Mycoplasma* testing was conducted every 2 months using the Mycoplasma PCR Detection Kit (Abcam). Cell line authentication was performed via short tandem repeat profiling through Laragen sequencing and genotyping.

### Immunoblotting

The following buffer was used to obtain whole cell lysates: Tris–HCL pH 7.4 (20 mmol/L), NaCl (150 mmol/L), EDTA (1 mmol/L), EGTA (1 mmol/L), Na3PO4 (2.5 mmol/L), NaF (5 mmol/L), Triton X-100 (1%), and glycerol (10%), supplemented fresh daily with a phosphatase inhibitor cocktail, 1% PMSF (Sigma-Aldrich), and protease inhibitor cocktail (Roche). The following antibodies (all from Cell Signaling Technologies) were used for Western blotting: c-Myc (#18583, RRID: AB_2895543), Mcl-1 (#94296, RRID: AB_2722740), cPARP (#9541, RRID: AB_331426), pAKTS473 (#4060, RRID: AB_2315049), Bcl-xL (#2764, RRID: AB_2228008), Bcl-2 (#4223, RRID: AB_1903909), Rpb1 (#2629, RRID: AB_2167468), pRbp1S2 (#13499, RRID: AB_2798238), pRbp1S5 (#13523, RRID: AB_2798246), β-tubulin (#5568, RRID: AB_10694505), and β-actin (#4970, RRID: AB_2223172).

### Cell viability assays and experimental compounds

Cell proliferation was quantified using a colorimetric tetrazolium-based assay. Cells were seeded in a 96-well plate at 15,000/well and treated with experimental compounds for 72 hours. CellTiter96 A_Queous_ One reagent (Promega) was added, and the optical density was measured at 490 nm after 4 hours. The IC_50_ value was calculated using nonlinear regression with a variable slope.

Apoptosis was quantified as described previously using the ApoScreen Annexin V Apoptosis Kit (1). Briefly, cells were suspended in Annexin V binding buffer containing 1 μL of Annexin V and 1 μL 7-aminoactinomycin D per 100 μL of binding buffer. To identify the B-cell population in primary samples, CD19 mAb (Southern Biotech) was added at the same dilution. After flow cytometry analysis on a LSRFortessa (BD Biosciences), data were analyzed with FlowJo software (Tree Star).

AZD4573 was purchased from ChemieTek, ibrutinib was from MedChemExpress, and IACS-010579 was from Selleck Chemicals.

### Functional analysis of mitochondria

Mitochondrial depolarization was quantified using the JC-1 MitoMP Detection Kit (Dojindo Molecular Technologies). After 48 hours incubation with drugs, cells were stained with JC-1 dye at 4 μmol/L concentration for 30 minutes, washed, resuspended in 1× imaging solution, and immediately analyzed by flow cytometry. Red indicates healthy polarized mitochondria (JC-1 aggregates), whereas green indicates loss of membrane potential (JC-1 monomers).

To quantify mitochondrial mass, 1.5 × 10^6^ cells/condition were treated with drugs for 48 hours in 12-well plates, washed, and incubated in 20 nmol/L MitoBright Green (Dojindo Molecular Technologies) and Live/Dead Aqua (Molecular Probes) for 30 minutes. Cells were washed and analyzed by flow cytometry. Dead cells (positive on Live/Dead Aqua stain) were excluded from analysis. MitoBright green mean fluorescence intensity was quantified, with lower intensity staining indicating a decrease in mitochondrial mass.

### Reactive oxygen species

Intracellular reactive oxygen species (ROS) levels were measured using the DCFDA/H2DCFDA Cellular ROS Assay kit (Abcam). Cells were incubated with DCFDA in RPMI medium for 30 minutes, washed and resuspended in 1 × 10^5^ cells/well in a 96-well black-walled, clear-bottomed plate (Brand), and incubated with drug for up to 48 hours. Fluorescence of the substrate was measured in SpectraMax iD3 (Molecular Devices).

### *In vivo* MCL models

All animal studies were approved by the Institutional Animal Care and Use Committee (IACUC) and were carried out in accordance with institutional guidelines (IACUC #20006). Six-week-old nonobese diabetic/severe combined immunodeficiency/γCnull [NOD.Cg-PrkdcscidIl2rgtm1Wjl/SzJ (NSG)] mice (The Jackson Laboratory, RRID: IMSR_JAX:005557) received 25 mg/kg busulfan via intraperitoneal injection. The following day, MCL cells (3 × 10^6^ in 200 μL PBS) were inoculated via tail vein injection. Two aggressive patient-derived xenograft (PDX) models were used: MCL44685 (ATMV1671fs, WHSC1E1009K, CREBBPQ2257H; further referred to as MCL-01) and MCL96069 (BIRC3L548fs; further referred to as MCL-02). Circulating MCL cells are detected within 2 weeks (MCL-01) and 3 weeks of inoculation (MCL-02). If left untreated, mice succumb to the disease within 6 and 9 to 10 weeks, respectively.

Mice were bled weekly, and blood was analyzed by flow cytometry to detect circulating MCL cells; red cells were lysed using ACK lysis buffer (Gibco), and the remaining cells were washed and stained with 0.5 μL LIVE/DEAD Fixable Aqua (Invitrogen), 20 μL CD19 (Becton Dickinson), 2 μL CD5, and 10 μL CD45 (Miltenyi Biotech) in 250 μL PBS and then analyzed by flow cytometry. MCL cells were classified as CD45^+^ lymphocytes that coexpressed CD5 and CD19. Upon detection of circulating cells, mice were separated into groups and received treatment with AZD4573 at 15 mg/kg either once or twice weekly diluted in 2% N,N-dimethylacetamide (DMAc), 30% PEG-400, and 68% of 1% (v/v) Tween-80 intraperitoneally, ibrutinib 25 mg/kg diluted in 1% 2-hydroxypropyl-β-cyclodextrin via oral gavage, or vehicle control [2% DMAc, 30% PEG-400, 68% (1%) Tween-80] intraperitoneally. [MCL01 = (control *n* = 4, AZD4573 1x weekly *n* = 11, AZD4573 2x weekly *n* = 9, ibrutinib *n* = 3) MCL02 = (control = 3, AZD4573 1x weekly *n* = 5, AZD4573 2x weekly *n* = 5, ibrutinib *n* = 5)].

Mice were followed for survival and were euthanized if weight loss exceeded 20%. At the time of sacrifice, spleen was homogenized via passage through a 40-μm cell strainer. Splenocytes were subjected to red blood cell lysis.

### Bulk RNA sequencing

Mouse splenocytes were washed, and total RNA was isolated using the E.Z.N.A. Total RNA Isolation Kit (Omega Bio-tek). RNA concentration was determined using the Qubit RNA HS Assay Kit (Invitrogen). A total of 2,500 ng of total RNA per sample (100 ng/μL) was submitted to Novogene Corporation. RNA integrity was determined using the 2100 Bioanalyzer Instrument (Agilent). Library preparation and sequencing were carried out by Novogene Corporation.

Samples were sequenced 12 per lane on a NovaSeq 6000 (Illumina). Partek Flow was utilized for data analysis. Trimming of adapters and bases for Fastq files was performed with Partek software. Mapping to the reference index hg38 was performed with STAR. Counts were then normalized to 10,000 counts per million. Gene set enrichment analysis (GSEA) was performed using Hallmark pathways (14, 15).

### Single-cell RNA sequencing

Following approval by the Institutional Review Board and with written informed consent obtained from patients, peripheral blood mononuclear cells (PBMC) were obtained from patients with MCL treated at the City of Hope National Medical Center (clinicaltrials.gov identifier: NCT04630756). Studies were conducted in accordance with the Declaration of Helsinki. For RNA analysis, peripheral blood was collected from patients treated with AZD4573 at the prespecified time points noted above. PBMCs were isolated using standard Ficoll-Hypaque technique (Amersham). Red blood cells were lysed using ACK buffer (Thermo Fisher Scientific). PBMCs were viably frozen for subsequent analysis.

### Single-cell library preparation and sequencing

Upon thawing, PMBCs were washed, and single cells were labeled with cell multiplexing oligos (CellPlex Kit Set A, 1000261; 10x Genomics) according to the manufacturer’s instructions. Labeled samples were pooled in desired ratios. Cell concentration and viability of the pooled sample was determined using the TC20 Automated Cell Counter (Bio-Rad). A total of 10,000 cells were targeted per pooled sample. Each sample pool was loaded into a different lane of a 10× chip (Chromium Next GEM Chip G Single Cell Kit, 1000127). cDNA libraries were generated using the Single Cell 3′ Library & Gel Bead Kit version 3.1 (1000121). Indexed sequencing libraries were constructed using the reagents in the library construction kit (10x Genomics, 1000190). The barcode sequencing libraries were sequenced on the NovaSeq 6000 platform (Illumina) with paired-end sequencing and dual indexing. A total of 28, 10, 10, and 101 cycles were run for read 1, i7 index, i5 index, and read 2, respectively. Raw sequences were processed and aligned to the GRChg38 genome using 10x Genomics’ Cell Ranger v6.1.1 “multi” pipeline with default settings. These experiments were performed in the Integrative Genomics and Bioinformatics Core and supported by the National Cancer Institute of the National Institutes of Health under award number P30CA033572.

### Data processing and quality control

Cell Ranger (v7.1.0) was used to demultiplex the samples and align the FASTQ files to the GRCh38 reference genome, generating filtered gene expression matrices. For downstream analysis, the filtered matrix files were processed using Seurat v5.1.0 (16). Samples from the same patient were merged into a single dataset while retaining metadata containing original sample annotations. Each individual sample underwent preprocessing steps, including normalization, scaling, and quality control. Quality metrics such as the number of detected genes, unique molecular identifier counts, and the percentage of mitochondrial gene expression were evaluated for each cell. Outlier cells were filtered out using stringent thresholds: cells with fewer than 550 or more than 8,500 unique features and cells with greater than 18% mitochondrial RNA content were excluded.

### Clustering and visualization

Cell clustering was performed using FindNeighbors and FindClusters functions, with the resolution parameter set to 0.5 (17). Dimensionality reduction and visualization were conducted using the RunUMAP function in Seurat. The resulting clusters were used as the foundation for downstream analyses, including biomarker identification and differential expression comparisons between clusters.

### Pathway enrichment analysis

To explore biological pathways enriched across cell types and time points, we performed GSEA. GSEA evaluates whether predefined gene sets exhibit statistically significant and coordinated differential expression between biological states, focusing on pathway-level changes rather than individual genes. For each comparison, differentially expressed genes (DEG) with log fold change (log FC) >0.2 and min.pct >0.05 were ranked based on both log FC and *P* values. The normalized enrichment scores (NES) were compared across the 50 hallmark gene sets, highlighting pathways with *P* value < 0.05.

Additionally, for specific pathways of interest, the AddModuleScore function was used to calculate pathway-specific gene group expression scores at the single-cell level. This enabled further quantification and visualization of pathway activity across distinct cell populations.

### Statistical analysis

All experiments were carried out with a minimum of three biological replicates unless otherwise noted. Statistical analysis was performed using the two tailed Student *t* test in GraphPad Prism (version 9.1.0). *, *P* < 0.05 and **, *P* < 0.01 throughout the article. Drug combination effect and synergistic score were calculated for the Zip independence dose–response surface model using R_SynergyFinder software (18).

## Results

### CDK9i restricts proliferation of ibrutinib-resistant MCL cells

**Figure 1.**
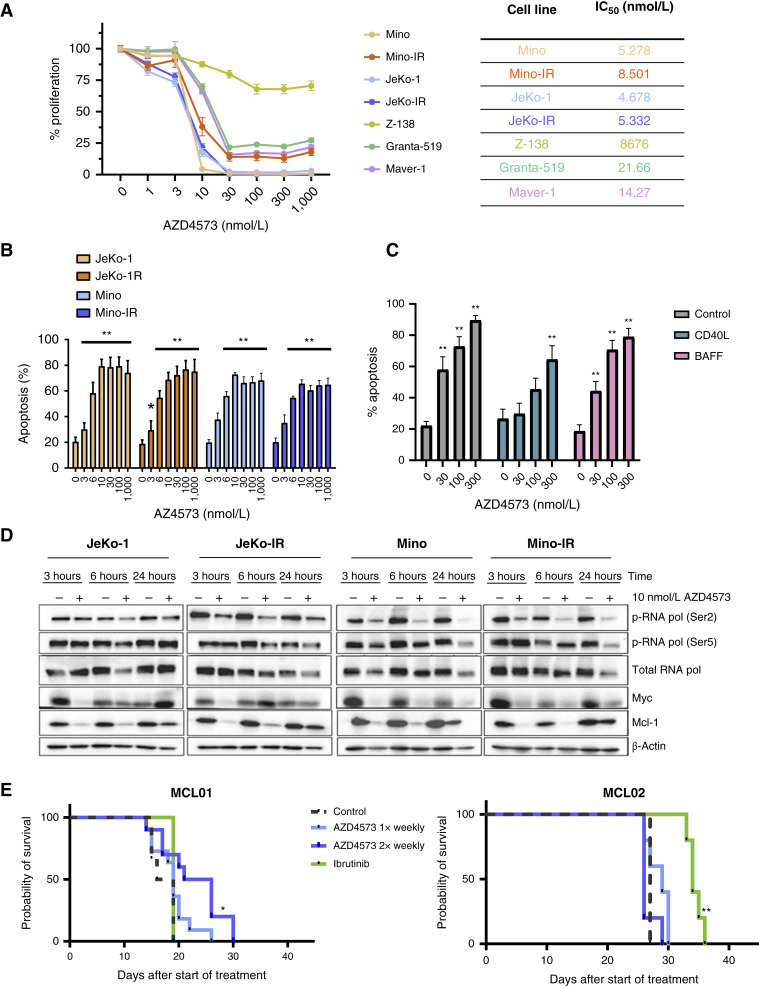
AZD4573 induces apoptosis and thwarts survival of MCL cells. **A,** MCL cell lines were treated with the indicated doses of AZD4573 for 72 hours. Cell proliferation was assessed using a colorimetric tetrazolium-based assay. The mean ± SEM is shown. IC_50_ value was calculated using GraphPad Prism software set to variable slope. **B,** Cells were treated with indicated doses of AZD4573 for 8 hours. Apoptosis was determined using Annexin V–FITC staining. Data are the mean ± SEM. *, *P* < 0.05 and **, *P* < 0.01 vs. untreated control. **C,** Primary MCL cells (*n* = 5 individual samples tested in technical triplicates) were cocultured with stromal cells for 24 hours and then treated with indicated doses of AZD4573 for 24 hours. Apoptosis of the CD19^+^ B-cell population was quantified using Annexin V–FITC staining. *, *P* < 0.05 and **, *P* < 0.01 vs. untreated control. **D,** Parental and IR MCL cell lines were treated with 10 nmol/L of AZD4573 or vehicle control for the indicated time points. Cell lysates were subjected to immunoblotting. **E,** Mice were inoculated with MCL. Two distinct MCL PDX models were used. Once circulating CD5^+^/CD19^+^ MCL cells were detected in the peripheral blood, mice began treatment as indicated in the methods (*n* = 3–11 per group). Kaplan–Meier survival curves are shown, with significance determined by the log-rank test. *, *P* < 0.05; **, *P* < 0.01.

First, we evaluated *in vitro* antitumor activity of selective CDK9i using a panel of MCL cell lines. Parental JeKo-1 and Mino as well as Granta-519 and MAVER-1 cell lines exhibited sensitivity to AZD4573 with IC_50_ values of 4 to 22 nmol/L, whereas Z-138 exhibited primary resistance (Fig. 1A; Supplementary Fig. S1A and S1B). Both ibrutinib-resistant Mino (Mino-IR) and Jeko-1 (JeKo-IR) cells retained sensitivity to CDK9i. Treatment with AZD4573 induced apoptosis of Jeko-1 and Mino cell lines in a dose-dependent manner regardless of ibrutinib sensitivity (Fig. 1B). Next, we assessed primary peripheral blood–derived MCL cells. To mimic the lymph node microenvironment, CD40L- and BAFF-expressing stroma was used, which we have previously shown to abrogate spontaneous and drug-induced apoptosis and confer ibrutinib resistance (19). We found that AZD4573 effectively induced apoptosis in primary peripheral blood–derived MCL cells under all stromal conditions (Fig. 1C).

Next, we evaluated the effect of AZD4573 on RNAPII using 10 nmol/L (approximate IC_50_) concentration. Consistent with known mechanism of action, CDK9i resulted in rapid loss of RNAPII phosphorylation at Ser2 (within 3 hours; Fig. 1D). We also observed a late decrease in RNAPII^Ser5^ phosphorylation (at 24 hours). As expected, CDK9i rapidly downmodulated MYC and MCL1 protein levels in both parental and ibrutinib-resistant MCL cells. However, this downregulation was transient, and we observed recovery of MYC expression at 6 and 24 hours, most prominently in JeKo-1/JeKo-IR cells (Fig. 1D).

To understand AZD4573’s effect *in vivo* we utilized two distinct MCL PDX models. In the MCL01 model, treatment was initiated when circulating MCL cells became detectable around 3 weeks after inoculation. Ibrutinib did not lead to prolongation of survival in this model (Fig. 1E). Once-weekly treatment with AZD4573 had a nonsignificant effect on survival. Meanwhile, a twice-weekly regimen resulted in increased survival by 6 and 5 days versus control- and ibrutinib-treated mice, respectively (*P* = 0.04). In the MCL02 model, circulating blasts were seen around 4 weeks after inoculation when treatment began. In this model, ibrutinib resulted in survival prolongation; however, tumors showed primary resistance to AZD4573 (Fig. 1E).

### CDK9i upregulates oxidative posphorylation in ibrutinib-resistant MCL cells

**Figure 2.**
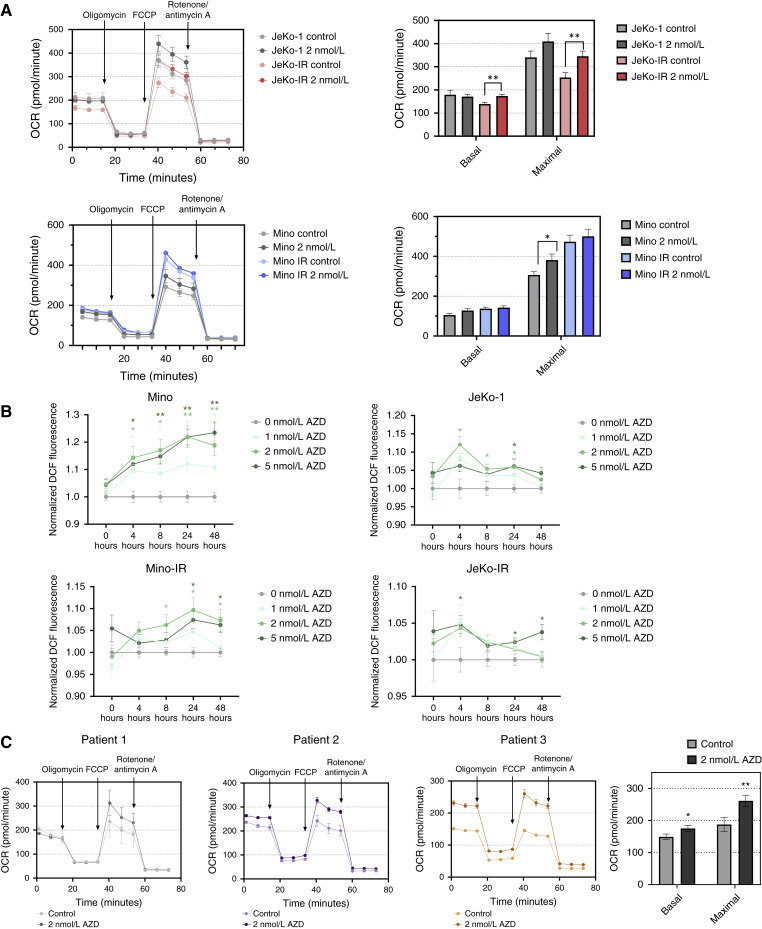
CDK9i leads to upregulated OxPhos. **A,** Cells were treated with either 2 nmol/L AZD4573 or control for 48 hours and then subjected to Seahorse analysis, utilizing the Mito Stress Test assay. The OCR was measured as a function of time with exposure to the inhibitors added at the indicated times: oligomycin (1.5 μmol/L), FCCP (0.5 μmol/L), and rotenone/antimycin A (0.5 μmol/L; *n* = 4 runs tested in quadruplicate) *, *P* < 0.05 and **, *P* < 0.01 vs. untreated control. **B,** Cells were stained with DCFDA and treated with indicated doses of AZD4573 (plated in triplicate). ROS mean fluorescence intensity quantification was taken at the indicated time points. *, *P* < 0.05; **, *P* < 0.001 vs. untreated control. **C,** MCL cells were first cocultured with CD40L-expressing stroma for 24 hours, then treated with 2 nmol/L AZD4573 for 24 hours, and then subjected to Seahorse analysis, utilizing the Mito Stress Test assay. The parameters are the same as in **A**. (*n* = 3 patients tested in quadruplicate) *, *P* < 0.05 and **, *P* < 0.01 vs. untreated control.

As oxidative posphorylation (OxPhos) upregulation has been implicated in ibrutinib resistance in MCL (20), we sought to further explore the effects of CDK9i on metabolic activity of ibrutinib-resistant MCL cells. We previously observed that in DLBCL cells, prolonged CDK9i (24 hours) led to recovery of oncogenic transcription (including *MYC*), ultimately contributing to drug resistance (12). Using the Seahorse assay, we quantified the oxygen consumption rate (OCR) at baseline and over time in response to inhibitors of the electron transport chain and OxPhos. We noted that basal OCR was notably higher in JeKo-1 than Mino cells (Fig. 2A). Both parental and ibrutinib-resistant JeKo-1 and Mino cells were treated with 2 nmol/L AZD4573 for 48 hours (higher concentrations resulted in excessive apoptosis over time). Such prolonged exposure to AZD4573 led to downmodulated MYC protein in the parental cells but not in the ibrutinib-resistant cells (Supplementary Fig. S2A). Treatment with AZD4573 increased basal OCR in JeKo-IR cells versus control-treated cells (174 and 139 pmol/minute, correspondingly), whereas this increase was not significant in Mino-IR cells (Fig. 2A; Supplementary Fig. S2B). Similarly, we observed an increase in the maximal OCR in AZD4573-treated JeKo-IR cells versus control-treated cells (345 vs. 254 pmol/minute, correspondingly), as well as in parental Mino cells, and a trend toward an increase in Mino-IR cells treated with AZD4573. We also observed an increase in ATP production in AZD4573-treated cells correlating with OCR increase (Supplementary Fig. S2C). OxPhos leads to ROS production. CDK9i resulted in a dose-dependent increase in ROS production in all tested cell lines, evident as early as 4 hours after treatment and sustained for up to 48 hours (Fig. 2B). To rule out increased cell proliferation as a cause for increased OxPhos, we performed Ki-67 staining in AZD4573-treated cells and observed no notable difference in any of the cell lines (Supplementary Fig. S3A).

We next investigated the effect of CDK9i on mitochondrial synthesis as a possible cause of increased OxPhos. MitoBright Green assay revealed no significant changes in mitochondrial mass following CDK9i (Supplementary Fig. S3B). Similarly, no alterations were observed in mitochondrial depolarization following the prolonged AZD4573 exposure independent of dose (Supplementary Fig. S3C). However, we observed a significant increase in mitochondrial depolarization in JeKo-IR cell lines compared with their parental counterparts, consistent with their higher OCR levels (Supplementary Fig. S3C).

Next, we quantified the metabolic activity of AZD4573-treated primary MCL cells. MCL cells isolated from patients were cocultured with stromal cells and treated with 2 nmol/L AZD4573. Due to the limited viability of primary cells in long-term *in vitro* cultures, we used shorter drug incubations (24 hours). Using Seahorse assay, we confirmed a significant increase in both basal and maximal OCR in the AZD4573-treated samples as compared with control, further validating our findings (Fig. 2C).

Thus, CDK9i results in upregulation of MCL cell metabolic activity, a mechanism which may account for subsequent resistance.

### CDK9i deregulates oncogenic pathways in MCL *in vivo*

**Figure 3.**
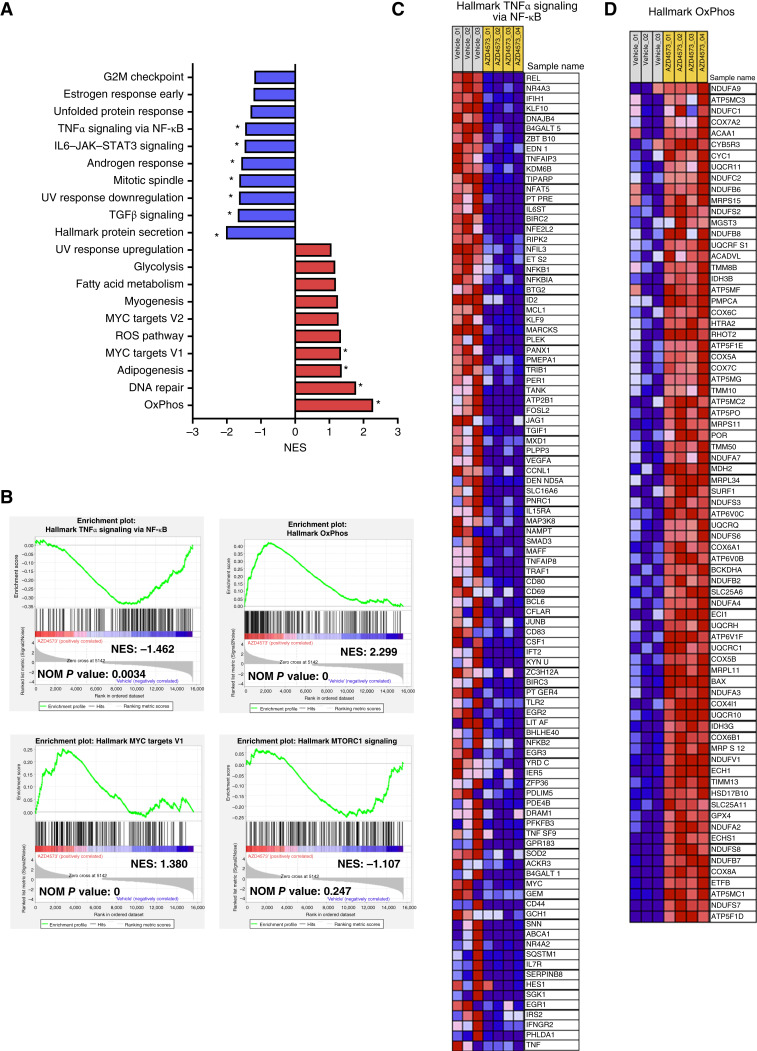
Pathway analysis of murine MCL splenocytes. **A,** GSEA of spleen-resident tumor cells from MCL01 mice treated with high-dose AZD4573 (2× weekly at 15 mg/kg) and vehicle that were subjected to RNA-seq analysis. Graph shown represents the top 10 hallmark pathways that were positively enriched and top 10 hallmark pathways that were negatively enriched in the AZD4573- vs. vehicle-treated mice. (* indicates nominal *P* value ≤ 0.05). **B,** GSEA plots for select gene sets. **C** and **D,** Heatmap depicting the ranked list of genes that had core enrichment for the TNFα signaling via NF-κB pathway and OxPhos as seen in **B**. Top row indicates treatment. Gray, three control-treated mice. Orange, four AZD4573-treated mice The range of expression values are visualized as red (high) to blue (low).

To gain further insight into potential mechanisms of resistance to CDK9i, we performed RNA sequencing (RNA-seq) on tumor splenocytes isolated from ibrutinib-resistant MCL01 mice which had received treatment with AZD4573 15 mg/kg twice weekly (*n* = 5) or vehicle control (*n* = 3) and despite this manifested progressive disease at the end of the experiment at the time of tissue collection (Fig. 1E). Using a cutoff of at least 1.5/−1.5-fold change, we identified 6,438 genes that were differentially expressed in the AZD4573-treated mice as compared with the vehicle control mice (*P*adj. < 0.05). Of those genes, 1,936 were upregulated and 4,502 were downregulated in the AZD4573 group. GSEA of the most significantly differentiated genes using the Hallmark database provided a list of deregulated pathways. TNFα signaling, TGFβ signaling, and IL6–JAK–STAT signaling were enriched among the most downregulated pathways, whereas OxPhos, MYC targets V1, and DNA repair were upregulated in murine tumors exposed to AZD4573 (Fig. 3A).

We observed that CDK9i abrogated the NF-κB pathway. TNFα signaling via NF-κB was among the most significantly negatively enriched pathways in AZD4573-treated mice (Fig. 3B). There were 97 core enrichment genes within this pathway (Fig. 3C). Of those, *REL* was the most significantly downregulated gene within the AZD4573-treated group along with *BIRC2*, *NFKB1*, *JUNB*, and other known NF-κB transcriptional targets. Consistent with known effects of CDK9i and earlier *in vitro* data, *MCL1* mRNA transcript was downregulated by AZD4573 in our *in vivo* model at the end of treatment (Fig. 3C).

Of the upregulated pathways, OxPhos was the most significantly affected (NES = 2.299; NOM *P* value = 0) in the AZD4573-treated mice compared with the control mice. The leading-edge subset within the OxPhos pathway was represented by the 75 core enrichment genes, many of which were related to components of the electron transport chain (Fig. 3D). Additionally, *MYC* targets V1 and V2 pathways were upregulated in AZD4573-treated mice.

Thus, consistent with *in vitro* data, *in vivo* exposure to AZD4573 led to persistent tumors which exhibited upregulation of the OxPhos pathway.

### AZD4573 downregulates oncogenic pathways in MCL cells in a clinical trial

We evaluated the tumor-intrinsic effects of AZD4573. Although we acknowledge the critical role of the lymph node niche on NHL cells, it was not feasible to procure serial specimens from that compartment. PBMCs were collected from patients at baseline, 4 and 24 hours (baseline and 7 hours for the patient with DLBCL) after AZD4573 infusion, and 7 days later (prior to administration of the subsequent weekly dose of AZD4573, which occurred on C1D8). Paired samples from the three patients were submitted for single-cell RNA-seq. Patient AZ01 had MCL with a high circulating MCL cell count in peripheral blood, which improved with AZD4573 treatment and was accompanied by reduction in splenomegaly, thus manifesting sensitivity to AZD4573 (Supplementary Fig. S4). Meanwhile, the second patient with MCL (AZ06), and a patient with DLBCL transformed from chronic lymphocytic leukemia (AZ04), showed no circulating malignant cells, and both patients exhibited resistance to AZD4573, as evidenced by disease progression on imaging after 8 weeks of treatment.

**Figure 4.**
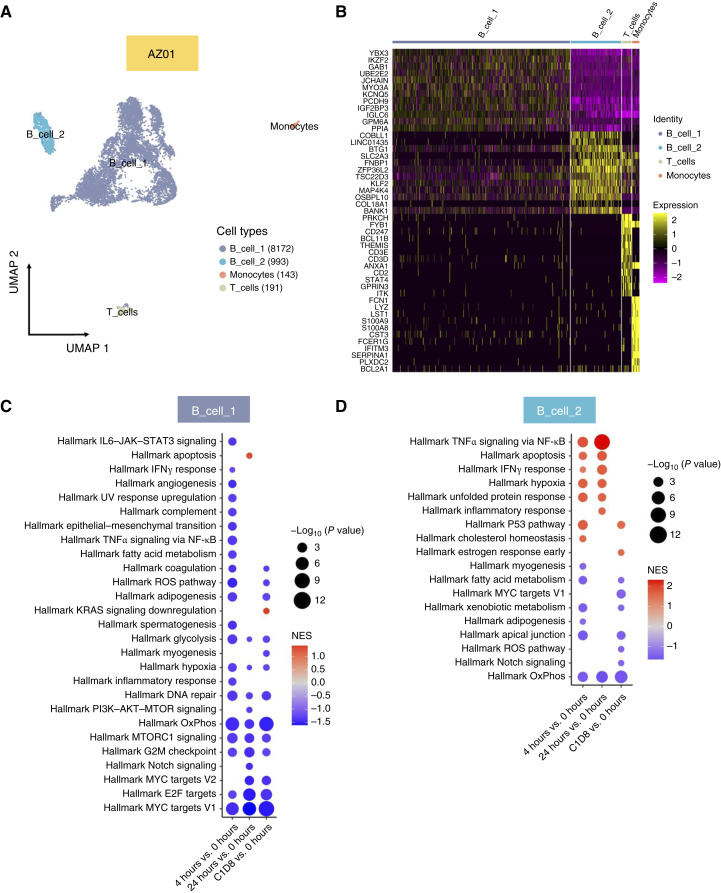
Single-cell RNA-seq analysis of PBMCs from a patient with MCL responsive to AZD4573 treatment on a clinical trial (AZ01). **A,** Uniform Manifold Approximation and Projection (UMAP) plot representation of integrated total PBMCs collected from the patient across four designated time points (*n* = 9,499 in total; baseline 0 hours, 4,663 cells; 4 hours, 859 cells; 24 hours, 2,666 cells; and C1D8, 1,311 cells) revealed four main cell populations. Population identities were determined based on marker gene expression. **B,** Normalized genes expression level heatmap of top marker genes per cluster. **C** and **D,** Dot plots showing the pathway enrichment on hallmark gene sets against differential expressed genes between time points, in B_cell_1 and B_cell_2 clusters, respectively. The ranks of genes per comparison group were calculated based on log FC and *P* value.

We further concentrated on the effect of AZD4573 on malignant B cells in the sensitive sample AZ01. After quality control, we obtained 9,499 cells (median number of genes per cell – 2,962; range, 554–8,286). In total, we identified four cell clusters: B_cell_1, B_cell_2, monocytes, and CD4^+^ T cells (Fig. 4A). No natural killer or myeloid cells were detected in this sample. We observed no clustering by time points (Supplementary Fig. S5A), suggesting negligible batch effect with multiplexing strategy. We identified two distinct B-cell clusters, exhibiting distinct gene expression patterns (Supplementary Table S1), with the top 10 most DEGs of each cluster visualized in the heatmap (adj.*P* < 0.05; Fig. 4B). Using inferCNV software with B cells from normal PBMCs as a reference, we assessed the copy-number variation profiles of various cell types of AZ01. Our analysis revealed a high-observation region on chromosome 7 in B-cell cluster 1, which was absent in B-cell cluster 2. Thus, we identified two types of malignant B cells, suggesting clonal heterogeneity in this sample. We focused on these B-cell clusters in our subsequent analysis.

**Figure 5.**
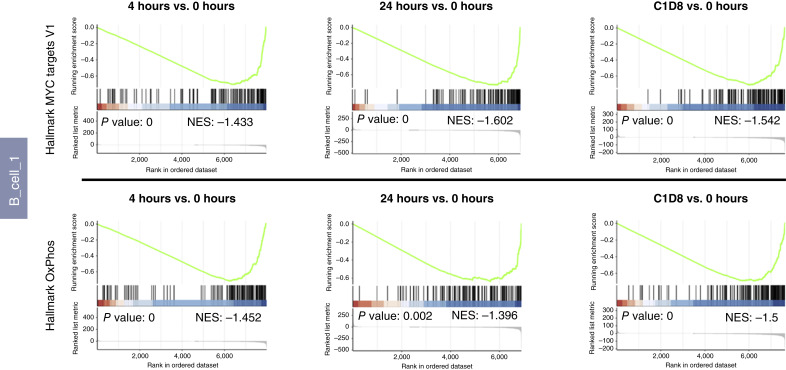
GSEA analysis of a patient sensitive to AZD4573 treatment. GSEA plots for hallmark MYC targets V1 and OxPhos gene sets for patient AZ01. Graphs shown are only for B_cell_1 cluster at the designated time points vs. 0 hour.

We first evaluated genes deregulated by AZD4573 treatment in the two B-cell clusters (Fig. 4C and D). B-cell cluster 1 had a higher number of significantly downregulated genes following treatment with AZD4573. We found downregulation of MYC targets and OxPhos in both B-cell clusters upon treatment with AZD4573, which was detected 4 hours after infusion and was sustained at all time points (Figs. 4C, D, and 5; Supplementary Fig. S5B and S5C). As noted previously in our mouse experiments, in this dataset we also saw early (4 hours) downregulation of TNFα signaling via NK-κB and IL6–JAK–STAT signaling. We also observed downmodulation of mTORC1 signaling and E2F targets in B-cell cluster 1, consistent with the expected mechanistic effects of CDK9i. Interestingly, certain gene sets (notably, NF-κB signaling–related genes) were upregulated in B-cell cluster 2 following treatment with AZD4573, suggesting differential subclonal response to CDK9i in MCL.

**Figure 6.**
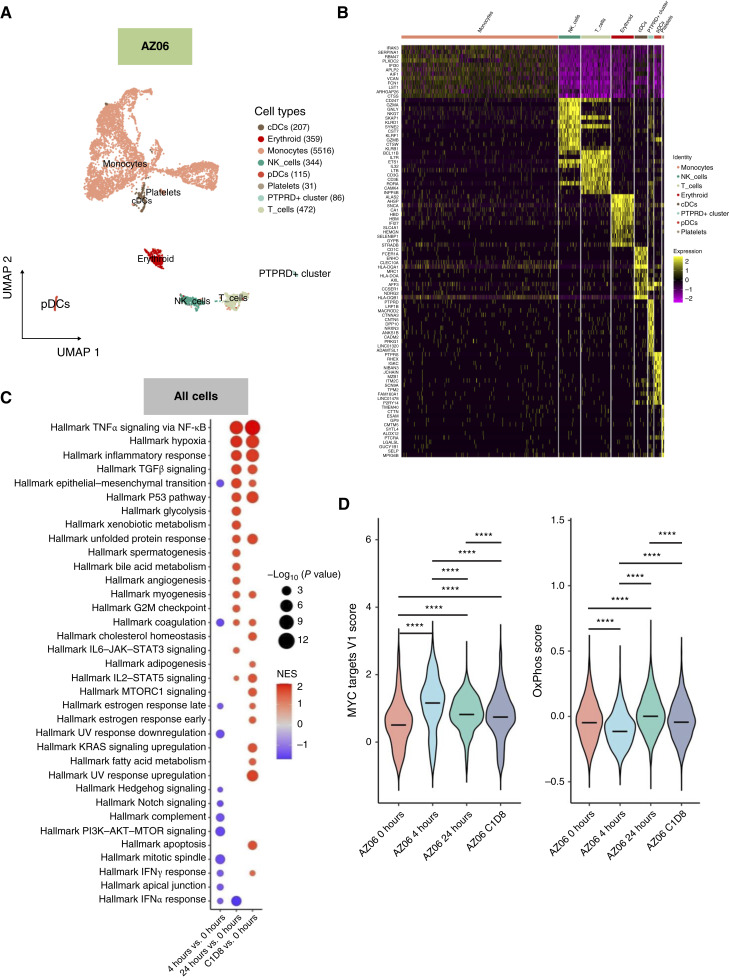
Single-cell RNA-seq analysis of PBMCs from patient refractory to AZD4573 treatment on a clinical trial (AZ06). **A,** Uniform Manifold Approximation and Projection (UMAP) plot representation of integrated total PBMCs collected from the patient across four designated time points (*n* = 7,130 in total; baseline 0 hour, 2,617 cells; 4 hours, 552 cells; 24 hours, 1,332 cells; and C1D8, 2,629 cells) identified eight main cell populations and associated markers. **B,** Normalized genes expression level heatmap of top marker genes per cluster. **C,** Dot plots showing the pathway enrichment on hallmark gene sets against differential expressed genes between time points in monocytes including all cells populations. The ranks of genes per comparison group were calculated based on log FC and *P* value. **D,** Violin plots of Hallmark MYC targets V1 and OxPhos gene sets scores (calculated by Seurat::AddModuleScore) across all cells at each time point, based on the enriched genes of each pathway. ****, *P* < 0.0001.

Meanwhile, monocytes were the predominant cell population in the AZ06 MCL sample (Fig. 6A). The top 10 most DEGs of each cluster are shown in the heatmap (*P*adj < 0.05; Fig. 6B). In contrast to AZ01 data, we observed upregulation of multiple oncogenic pathways in this resistant sample, including MYC targets, OxPhos, and NF-κB signaling (Fig. 6C and D). The third sample also revealed monocytes as the predominant cell population (Supplementary Fig. S6A). In this patient, treatment with AZD4573 resulted in initial downregulation of OxPhos, TNFα/NFκB, and other oncogenic pathways, followed by recovery of OxPhos and upregulation of MYC pathways on C1D8 (Supplementary Fig. S6B–S6D).

Thus, using primary samples from patients treated with AZD4573, we observed rapid deregulation of multiple genes, in particular downregulation of MYC and OxPhos signaling in the malignant B cells. This effect was sustained in a sensitive sample, whereas resistant samples demonstrated either lack of effect or rapid recovery of these pathways in nontumor PBMCs.

### Concurrent targeting of OxPhos enhances the antitumor effect of CDK9i

**Figure 7.**
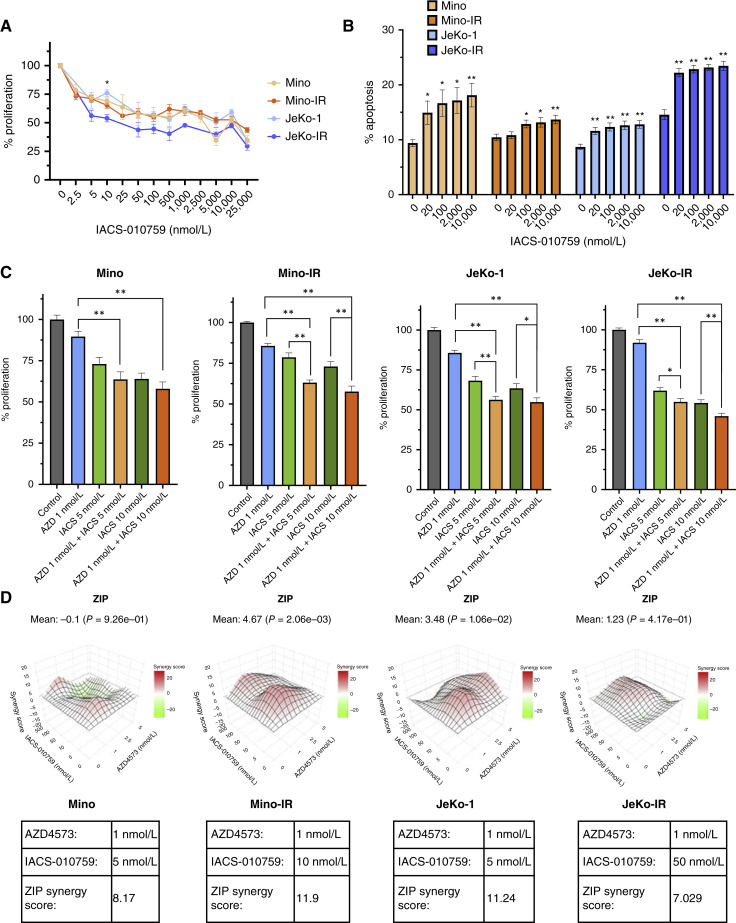
Concurrent CDK9 and OxPhos inhibition inhibits growth of MCL cells. **A,** Parental and ibrutinib-resistant MCL cell lines were treated with the indicated doses of IACS-010579 for 72 hours. Cell proliferation was assessed using a colorimetric tetrazolium-based assay. The mean ± SEM is shown. *, *P* < 0.05 for JeKo-1 vs. JeKo-1R comparison. **B,** MCL cell lines were treated with IACS-010579 at the indicated doses for 24 hours (*n* = 3 tested in duplicates). Apoptosis was determined by flow cytometry using Annexin V–FITC staining. *, *P* < 0.05 and **, *P* < 0.01 compared with control. **C,** MCL cell lines were treated with IACS-010579 and/or AZD4573 as single agents or in combination at the indicated doses for 72 hours. Cell proliferation was assessed using a colorimetric tetrazolium-based assay. The mean ± SEM is shown. *, *P* < 0.05 and **, *P* < 0.01 compared with control. **D,** 3D heat maps of synergy scores for cell lines treated with IACS-010759 and AZD4573 as seen in **C**. Tables show the combinations for each cell line with the highest ZIP synergy scores.

Given our findings, we next evaluated whether targeting of OxPhos with a small-molecule inhibitor might enhance CDK9i in MCL. IACS-010759, an effective inhibitor of complex I of the mitochondrial electron transport chain, has been studied in phase 1 clinical trials (21). Treatment with IACS-010759 had a modest effect on the proliferation or apoptosis of MCL cell lines, with highest activity registered in JeKo-IR cells (Fig. 7A and B; Supplementary Fig. S7). Furthermore JeKo-IR cells demonstrated higher sensitivity to IACS-010759 compared with parental JeKo-1 cells (IC_50_ of 1.1 μmol/L vs. 27.3 μmol/L), confirming that IR cells are more dependent on OxPhos (Supplementary Fig. S7).

Combined treatment with AZD4573 and IACS-010759 demonstrated cooperative effect on cell proliferation compared with either drug alone (Fig. 7C). The effect was additive in JeKo-IR cells (ZIP synergy score 7.03) and synergistic in Mino-IR cells (ZIP score 11.9 at 1 nmol/L AZD4573 and 10 nmol/L IACS-010759; Fig. 7D). However, a comparison of the effect of this combination between IR and parental cell lines did not produce significance, suggesting that the cooperative effect of concurrent targeting CDK9 and OxPhos is not unique to the BTKi-resistant setting.

## Discussion

BTKis have become a standard of care for the treatment of MCL. However, all patients inescapably develop resistance. Therapeutic strategies in patients with MCL who develop BTKi resistance are limited and include chimeric antigen receptor T cells (CAR-T), as well as investigational agents such as Bcl-2 inhibitor venetoclax and bispecific antibodies (22). Although the best efficacy may be achieved with CAR-T cells, emerging data suggest that BTKi-refractory patients have inferior outcomes compared with BTKi-naïve patients (8). In our study, we demonstrated that selective targeting of CDK9 with AZD4573 led to apoptosis of ibrutinib-resistant MCL cell lines and primary cells, suggesting that CDK9i may be effective in combating BTKi resistance. *MYC* oncogene is deregulated in MCL, and its overexpression is associated with inferior outcomes (23). Furthermore, MYC signature is suppressed by ibrutinib in BTKi-sensitive cells, but not in resistant cells, implicating MYC in intrinsic resistance to BTKis (24). We observed rapid, albeit transient, downmodulation of MYC protein in parental and ibrutinib-resistant MCL cells, consistent with our previous observations (12). In MCL PDX models, MYC targets were upregulated in residual tumors, further suggesting that CDK9i could not fully overcome MYC-driven resistance. Analysis of tumor cells from a patient with MCL treated with AZD4573 on a clinical trial showed downregulation of MYC-related pathways at earlier time points. Unfortunately, samples were not available at the time of progression to evaluate MYC expression. Analysis of PBMCs from patients refractory to AZD4573 on the same trial showed that MYC pathways were not inhibited, albeit tumor cells were not available for analysis in these patients.

We and others have previously shown that CDK9i downregulates NF-κB (25). NF-κB has also been implicated in BTKi resistance in MCL (26). Constitutive NF-κB activity may result from recurrent mutations in *TRAF2*, *BIRC3*, and *RELA* (26–28). Mutations in genes indirectly involved in regulation of NF-κB signaling were detected in primary ibrutinib-refractory MCL samples (29). Suppression of NF-κB, among other pathways, by pharmacologic targeting of MALT1, partially abrogated BTKi resistance in MCL (30). However, although we observed downregulation of NF-κB signaling pathways by AZD4573 *in vivo*, this was not sufficient to kill MCL. Furthermore, *BIRC3* gene mutations (which is present in MCL-02 model in our study) have previously been shown to lead to activation of the noncanonical NF-κB pathway in ibrutinib-resistant MCL cell lines (27, 31). Thus, this mutation may potentially act as an escape mechanism from CDK9i.

MYC plays an essential role in the regulation of metabolic reprogramming in cancer cells, enabling uncontrolled proliferation (32, 33). Activation of the mTOR–MYC–OxPhos pathway has been linked to proliferative disease in lymphoid tumors (34). OxPhos was one of the top upregulated pathways in AZD4573-resistant murine PDXs. CDK9i also led to activation of OxPhos *in vitro*, which was particularly prominent in ibrutinib-resistant cell lines and observed in primary MCL cells in stromal cocultures. Recent studies have shown that certain lymphoid cancers exhibit heightened dependency on OxPhos for energy production (35, 36). An increased dependency on OxPhos can be associated with therapeutic resistance (37, 38). Metabolic reprogramming toward OxPhos and glutaminolysis has previously been implicated in therapeutic resistance to ibrutinib in MCL (20). Our experiments show that CDK9i may heighten OxPhos, along with mitochondrial depolarization and ROS production (an OxPhos byproduct), suggesting that upregulation of OxPhos may be a global drug resistance mechanism in MCL. Targeting OxPhos with IACS-010759, an inhibitor of complex I of the mitochondrial electron transport chain, results in synergy with AZD4573 in MCL cells. Unfortunately, OxPhos inhibitors have not been successful in clinical trials thus far due to toxicity.

In sum, we demonstrate that CDK9i with AZD4573 results in decreased proliferation and apoptosis of ibrutinib-resistant cells and primary MCL cells in stromal conditions *in vitro* and exerts modest antitumor effect *in vivo*. Although we observe initial downregulation of MYC targets and other oncogenic pathways, including in a patient treated with AZD4573, this effect seems transient. Furthermore, prolonged exposure to AZD4573 leads to upregulation of OxPhos, which contributes to resistance to CDK9i. Thus, whereas CDK9 inhibitors may partially overcome ibrutinib resistance, they will need to be used as part of combination therapy approaches, such as with drugs that target OxPhos.

## Supplementary Material

Supplemental Figure 1 MCL cell lines treated with AZD4573 prolifereation assay

Supplemental Figure 2 Immunoblotting and Seahorse data for Mino and JeKo-1 cell lines

Supplemental Figure 3 Flow cytometry analysis for Ki67, MitoBright Green, and JC-1 in Mino and JeKo-1 cells.

Supplemental Figure 4 Weekly lymphocyte counts for patient AZ01

Supplemental Figure 5 Single cell sequencing data for patient AZ01

Supplemental Figure 6 Single cell sequencing data for patient AZ04

Supplemental Figure 7 Cell proliferation assay for MCL cell lines treated with IACS-010759

Supplemental Table 1 Single cell sequencing gene data

## Data Availability

The data generated in this study are available upon request to the corresponding author. High-throughput sequencing data were deposited in Gene Expression Omnibus (# GSE327598).
