# Supplementary material for: Cyclin-Dependent Kinase-9 and Oxidative Phosphorylation Inhibition Overcomes Ibrutinib Resistance in Mantle Cell Lymphoma
Source: Cancer Res Commun. 2026 May 22;6(5):1192–205. doi: 10.1158/2767-9764.CRC-25-0818 (PMC13195486; doi:10.1158/2767-9764.CRC-25-0818)
Supplement: Supplemental Figure 1 — MCL cell lines treated with AZD4573 prolifereation assay [file crc-25-0818_supplemental_figure_1_suppsf1.docx]

**Supplemental Figure 1**

# A

**Normalized Cell Viability (%)**

**125**


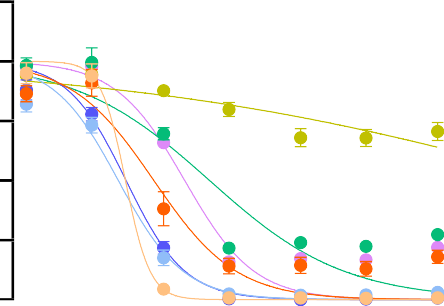


**100**

**75**

**50**

**25**

**0**

**0**

**1 2 3**

**Log [AZD4573] (nM)**


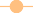
 Mino
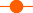
 Mino IR
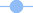
 JeKo-1


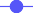
 JeKo-IR
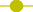
 Z-138


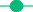
 Granta-519
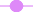
 Maver-1

**IC_50_ = 5.278 nM IC_50_ = 8.501 nM**

**IC_50_ = 4.678 nM IC_50_ = 5.332 nM IC_50_ = 8676 nM IC_50_ = 21.66 nM IC_50_ = 14.27 nM**

# B

**150**


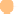


**% Proliferation**

**100**


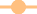
 Mino
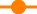
 Mino IR
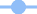
 JeKo-1
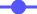
 JeKo-IR

**50**

**0**

**0 1 2 3 5 10 30 100**

**AZD4573 (nM)**

**Supplemental Figure 1**

(**A,B**) MCL cell lines were treated with the indicated doses of AZD4573 as seen in Figure 1B for 72 hours. Sigmoidal dose-response curve was generated by GraphPad prism software. Mean ± SEM as well as IC_50_ values to AZD4573 are shown.
