# Supplementary material for: Cyclin-Dependent Kinase-9 and Oxidative Phosphorylation Inhibition Overcomes Ibrutinib Resistance in Mantle Cell Lymphoma
Source: Cancer Res Commun. 2026 May 22;6(5):1192–205. doi: 10.1158/2767-9764.CRC-25-0818 (PMC13195486; doi:10.1158/2767-9764.CRC-25-0818)
Supplement: Supplemental Figure 2 — Immunoblotting and Seahorse data for Mino and JeKo-1 cell lines [file crc-25-0818_supplemental_figure_2_suppsf2.docx]

**Supplemental Figure 2**

# A


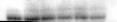

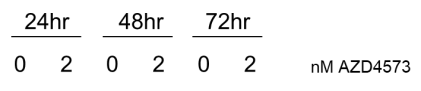

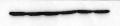


**Myc**

**β-Actin**


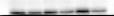

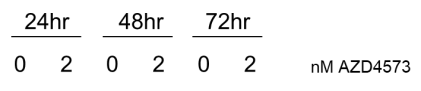


**Myc**

**Mino**

**Myc**


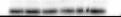


**Mino IR**

**β-Actin**


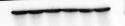


**β-Actin Myc**

**β-Actin**


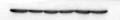

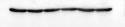

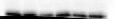


**JeKo-IR**

**JeKo-1**

# B

**800**

**600**

**OCR (pmol/min)**

**400**

**200**

**0**

**Mino**

**0 20 40 60 80**


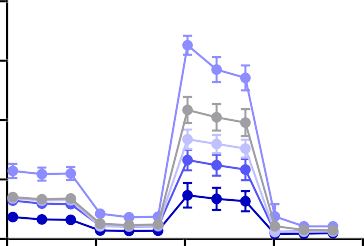


**Time (minutes)**

0nM AZD

1nM AZD

2nM AZD

5nM AZD

10nM AZD

**800**

**600**

**OCR (pmol/min)**

**400**

**200**

**0**

**Mino IR**


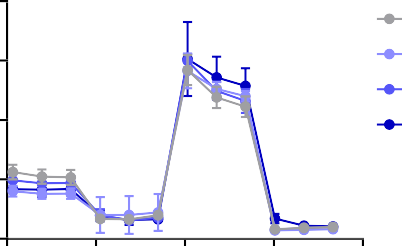


**0 20 40 60 80**

**Time (minutes)**

0nM AZD

1nM AZD

2nM AZD

5nM AZD

**800**

**600**

**OCR (pmol/min)**

**400**

**200**

**0**

# C

**150**

**JeKo-1**

**
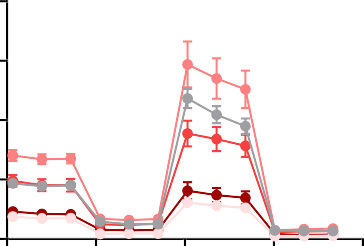
0 20 40 60 80**

**Time (minutes)**

**ATP Production**

**200**

✱✱


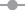
 0nM AZD


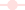
 1nM AZD


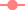
 2nM AZD


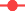
 5nM AZD


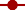
 10nM AZD

**ATP Production**

✱✱

**500**

**400**

**OCR (pmol/min)**

**300**

**200**

**100**

**0**

**JeKo-IR**

**
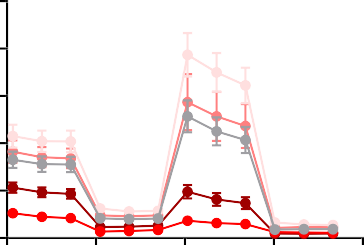
0 20 40 60 80**

**Time (minutes)**


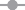
 0nM AZD


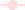
 1nM AZD


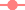
 2nM AZD


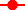
 5nM AZD


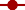
 10nM AZD

**100**

**OCR (pmol/min)**

**150**

**100**

**50**

**50**

**0 0**

**OCR (pmol/min)**


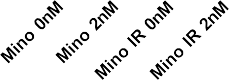

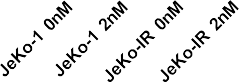


**Supplemental Figure 2**

**A** Cells were treated with either 2 nM AZD4573 or vehicle control for the indicated amount of time and subjected to immunoblotting.

**B,C** Cells were treated with either 1 nM, 2 nM, 5 nM, 10 nM AZD4573 or control for 48 hours and then subjected to Seahorse analysis, utilizing the Mito Stress Test Assay. The oxygen consumption rate was measured as a function of time with exposure to the inhibitors added at the indicated times: Oligomycin (1.5μM), FCCP (0.5μM), and rotenone/antimycin A (0.5μM). (n=1 run tested in quadruplicate)

**B** OCR graphs for JeKo-1/IR and Mino/IR cell lines.

**C** ATP Production measured as (Last rate measurement before Oligomycin injection) - (Minimum rate measurement after Oligomycin injection).
