# Supplementary material for: Cyclin-Dependent Kinase-9 and Oxidative Phosphorylation Inhibition Overcomes Ibrutinib Resistance in Mantle Cell Lymphoma
Source: Cancer Res Commun. 2026 May 22;6(5):1192–205. doi: 10.1158/2767-9764.CRC-25-0818 (PMC13195486; doi:10.1158/2767-9764.CRC-25-0818)
Supplement: Supplemental Figure 3 — Flow cytometry analysis for Ki67, MitoBright Green, and JC-1 in Mino and JeKo-1 cells. [file crc-25-0818_supplemental_figure_3_suppsf3.docx]

**Supplemental Figure 3**

# A

**15**


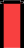

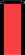

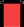

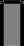

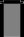

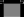


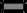
 0nM AZD


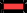
 2nM AZD

**% Ki-67+ cells**

**10**

**5**

**
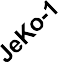

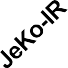

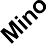

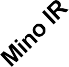
0**

**B 250**

**Normalized MFI of MitoBright Green**

**200**

**150**


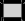
 0nM AZD


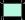
 1nM AZD


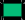
 2nM AZD


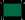
 5nM AZD

**100**

**50**

**0**

**Mino Mino IR JeKo-1 JeKo-IR**

# C

**25**


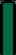

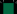

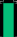

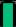

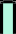

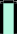

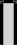

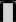

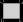

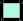

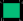


✱

**% Depolarization**

**20**

**15**

**10**

**5**

**0**

**Mino Mino IR JeKo-1 JeKo-IR**

0nM AZD

1nM AZD

2nM AZD

5nM AZD

**JeKo-IR**

**
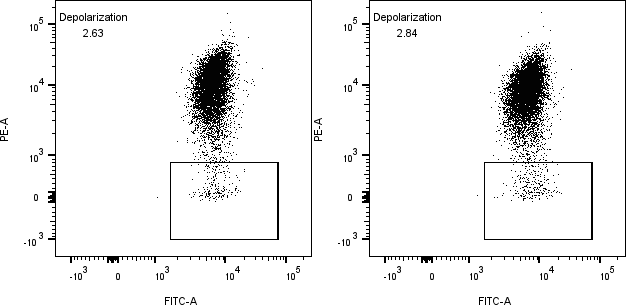
Control 2nM AZD4573**

**Supplemental Figure 3**

**A** Flow cytometry analysis measuring % Ki67+ cells of total live cells in JeKo-1/IR and Mino/IR cells after treatment with 2 nM of AZD4573 for 48 hours (n=3).

**B** MCL cell lines were treated with AZD4573 for 48 hours as indicated and mitochondrial mass was assessed using MitoBright Green via flow cytometry. A representative histogram is shown. Data are mean ± SE.

**C** Cells were treated with indicated doses of AZD4573 for 48 hours and then analyzed for mitochondrial depolarization with JC-1 dye using flow cytometry. A representative dot plot

image is shown (depolarized mitochondria is boxed).
