# Supplementary material for: Cyclin-Dependent Kinase-9 and Oxidative Phosphorylation Inhibition Overcomes Ibrutinib Resistance in Mantle Cell Lymphoma
Source: Cancer Res Commun. 2026 May 22;6(5):1192–205. doi: 10.1158/2767-9764.CRC-25-0818 (PMC13195486; doi:10.1158/2767-9764.CRC-25-0818)
Supplement: Supplemental Figure 4 — Weekly lymphocyte counts for patient AZ01 [file crc-25-0818_supplemental_figure_4_suppsf4.docx]

**Supplemental Figure 4**

**60000**


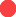

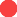

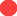

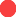

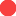

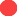


**Lymphocyte count (cells/μL)**

**40000**

**20000**

**0**

**0 1 2 3 4 5**

**Weeks on treatment**

**Supplemental Figure 4**

Weekly lymphocyte counts of patient AZ01 while on treatment with AZD4573.
