# Supplementary material for: Cyclin-Dependent Kinase-9 and Oxidative Phosphorylation Inhibition Overcomes Ibrutinib Resistance in Mantle Cell Lymphoma
Source: Cancer Res Commun. 2026 May 22;6(5):1192–205. doi: 10.1158/2767-9764.CRC-25-0818 (PMC13195486; doi:10.1158/2767-9764.CRC-25-0818)
Supplement: Supplemental Figure 5 — Single cell sequencing data for patient AZ01 [file crc-25-0818_supplemental_figure_5_suppsf5.docx]

**Supplemental Figure 5**

# A C

B cells 1

**AZ01**

**
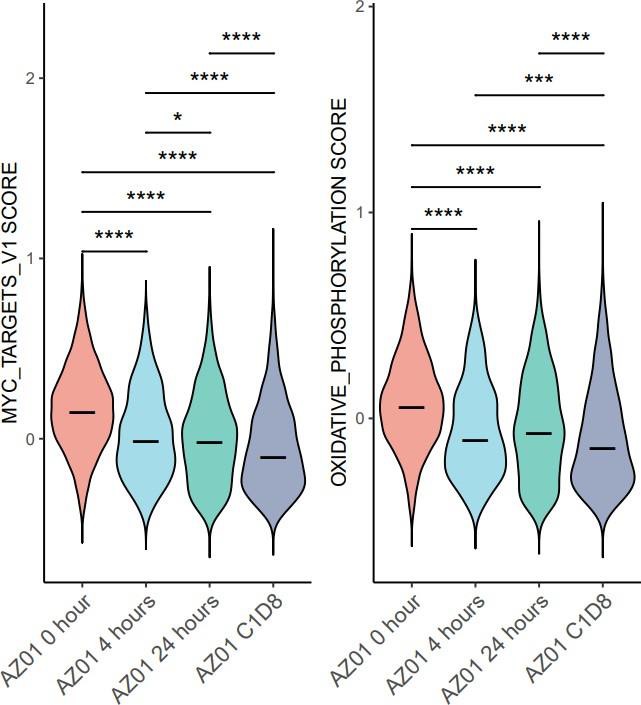
**


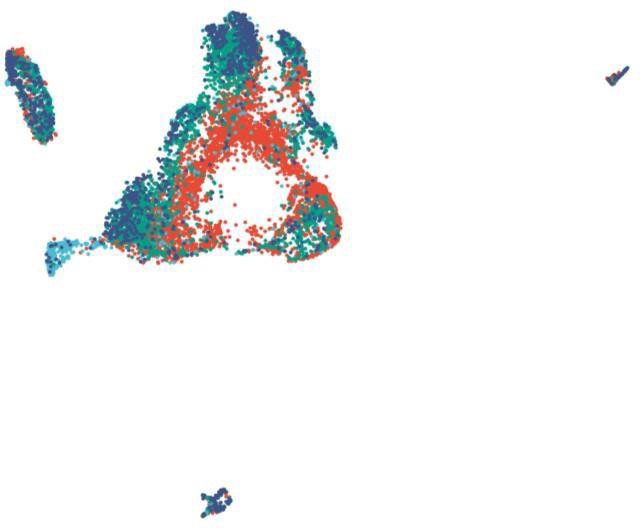

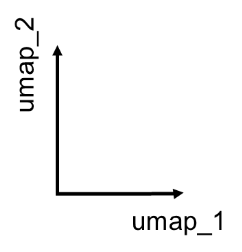

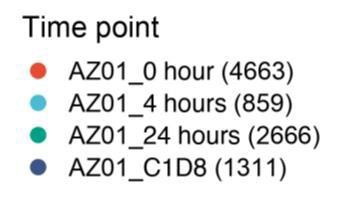


# B

**4 hours vs 0 hour 24 hours vs 0 hour C1D8 vs 0 hour**

B cells 2

**HALLMARK_MYC**

**_TARGETS_V1**

**
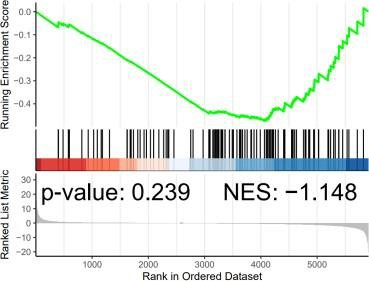

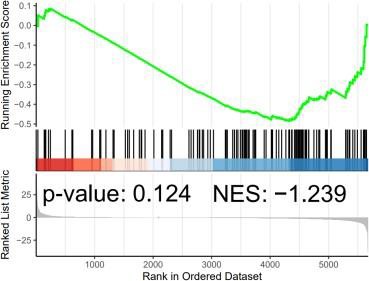

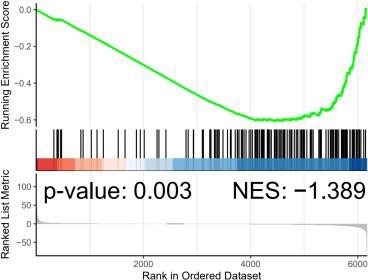
**

**4 hours vs 0 hour 24 hours vs 0 hour C1D8 vs 0 hour**

**AZ01**

**HALLMARK_OXIDATIVE_ PHOSPHORYLATION**


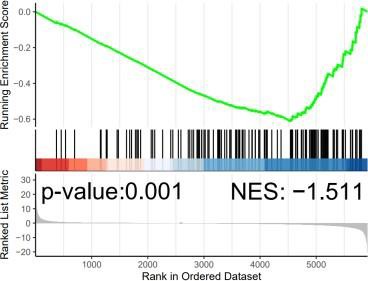

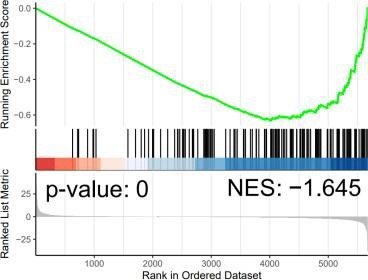

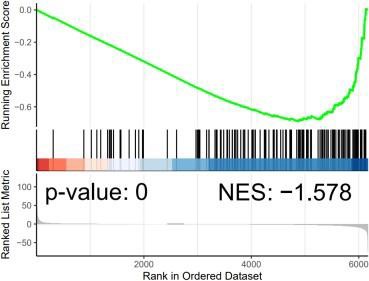


**Supplemental Figure 5**

**A** UMAP plot representation of integrated total PBMCs collected from the AZ01 patient across 4 designated time points (n=9,499 in total; baseline 0 hour, 4663 cells; 4 hours, 859 cells; 24 hours, 2666 cells; C1D8, 1311 cells).

**B** Gene set enrichment analysis plots for Hallmark MYC Targets V1 and Oxidative Phosphorylation gene sets. Graphs shown are only for B cells 2 cluster at the designatedtimepoints vs baseline 0 hour in AZ01.

**C** Violin plots of Hallmark MYC Targets V1 and Oxidative Phosphorylation pathway scores for the cells in AZ01 B cells 1 population at each time point. The scores were calculated based on enriched genes of each pathway.
