# Supplementary material for: Cyclin-Dependent Kinase-9 and Oxidative Phosphorylation Inhibition Overcomes Ibrutinib Resistance in Mantle Cell Lymphoma
Source: Cancer Res Commun. 2026 May 22;6(5):1192–205. doi: 10.1158/2767-9764.CRC-25-0818 (PMC13195486; doi:10.1158/2767-9764.CRC-25-0818)
Supplement: Supplemental Figure 6 — Single cell sequencing data for patient AZ04 [file crc-25-0818_supplemental_figure_6_suppsf6.docx]

**Supplemental Figure 6**


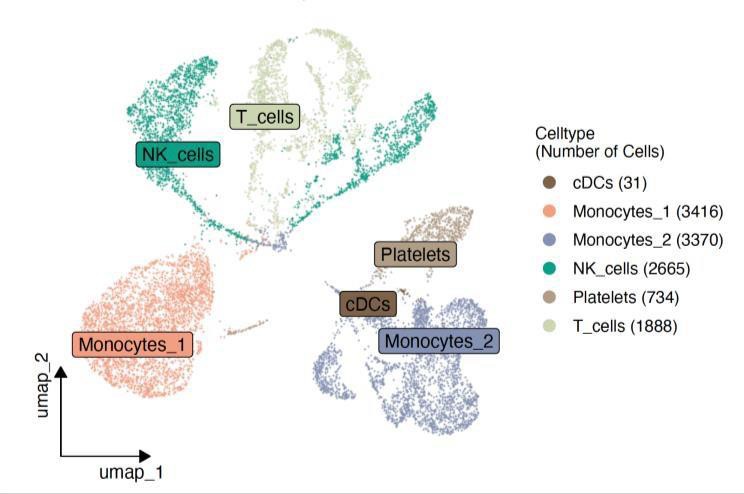

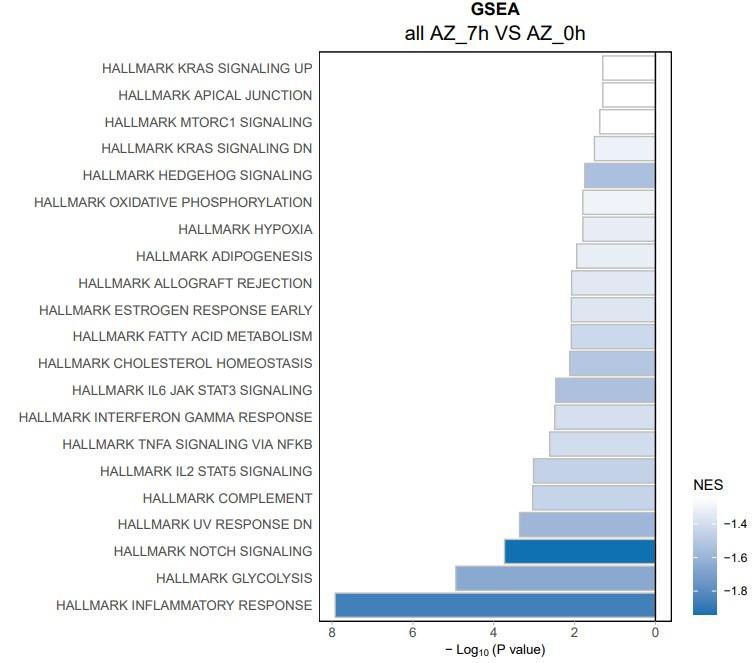


**A**

**AZ04**

**B**

# D


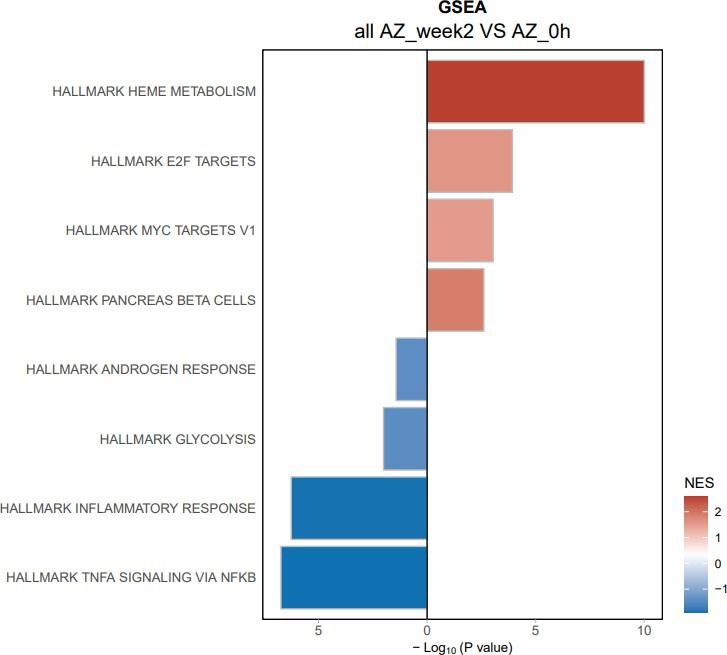

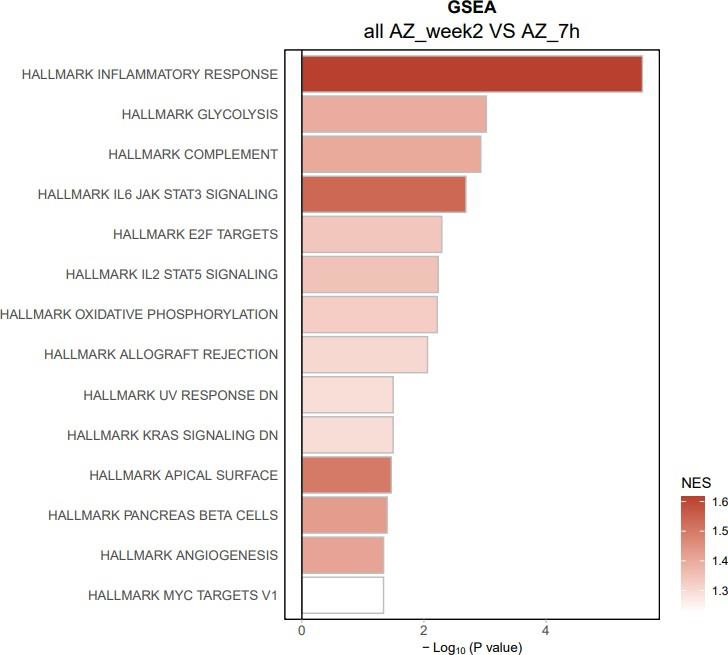


**C**

**Supplemental Figure 6**

Data are for a patient with DLBCL that was treated with AZD4573 on a clinical trial (labeled as AZ04).

**A** UMAP plot representation of total PBMC’s collected from the patient (n=1,2104 in total; baseline 0 hour, 3194 cells; 4 hours, 3647 cells; 24 hours, 3517 cells; C1D8, 1746 cells).

**B** Pathways downregulated in total cell population in 7 hours vs. 0 hour.

**C** Pathways upregulated in total cell population in C1D8 vs. 7 hours.

**D** Most significant pathways in total cell population in C1D8 vs. 0 hour.
