# Supplementary material for: Cyclin-Dependent Kinase-9 and Oxidative Phosphorylation Inhibition Overcomes Ibrutinib Resistance in Mantle Cell Lymphoma
Source: Cancer Res Commun. 2026 May 22;6(5):1192–205. doi: 10.1158/2767-9764.CRC-25-0818 (PMC13195486; doi:10.1158/2767-9764.CRC-25-0818)
Supplement: Supplemental Figure 7 — Cell proliferation assay for MCL cell lines treated with IACS-010759 [file crc-25-0818_supplemental_figure_7_suppsf7.docx]

**Supplemental Figure 7**

**100**

**% Proliferation**

**75**

**50**

**25**

**0**


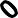

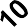


**
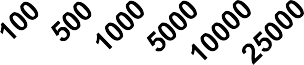
**

**IACS-010759 (nM)**


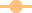
 Mino
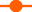
 Mino IR
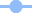
 JeKo-1


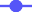
 JeKo-IR
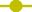
 Z-138


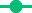
 Granta
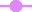
 Maver

**125**

**Normalized Cell Viability (%)**

**100**

**75**

**50**

**25**

**0**

**0**

**1 2 3**

**Log [IACS-010759] (nM)**


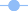
 JeKo-1
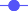
 JeKo-IR


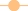
 Mino
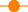
 Mino IR

IC_50_ = 27.3 μM IC_50_ = 1.13 μM IC_50_ = 3.99 μM IC_50_ = XX

**Supplemental Figure 7**

MCL cell lines were treated with the indicated doses of IACS010759 for 72 hours. Cell proliferation was assessed using a colorimetric tetrazolium-based assay. Sigmoidal dose-response curve was generated by GraphPad prism software. Mean ± SEMas well as IC50 values to IACS-010758 are shown.
